# WT1, p53 and p16 expression in the diagnosis of low- and high-grade serous ovarian carcinomas and their relation to prognosis

**DOI:** 10.18632/oncotarget.24530

**Published:** 2018-02-19

**Authors:** Luis Felipe Sallum, Liliana Andrade, Susana Ramalho, Amanda Canato Ferracini, Rodrigo de Andrade Natal, Angelo Borsarelli Carvalho Brito, Luis Otávio Sarian, Sophie Derchain

**Affiliations:** ^1^ Department of Obstetrics and Gynecology, State University of Campinas, Campinas, Faculty of Medical Sciences, Campinas, São Paulo, Brazil; ^2^ Department of Pathology, University of Campinas, Campinas, Faculty of Medical Sciences, Campinas, São Paulo, Brazil; ^3^ Program in Medical Sciences, State University of Campinas, Campinas, Faculty of Medical Sciences, Campinas, São Paulo, Brazil; ^4^ Laboratory of Investigative and Molecular Pathology, State University of Campinas, Campinas, Faculty of Medical Sciences, Campinas, São Paulo, Brazil; ^5^ Laboratory of Cancer Genetics, State University of Campinas, Campinas, Faculty of Medical Sciences, Campinas, São Paulo, Brazil

**Keywords:** cystadenocarcinoma, serous, diagnoses, survival, prognosis, Pathology

## Abstract

**Objective:**

To evaluate the diagnostic and prognostic value of the immunohistochemical expression of WT1, p53 and p16 in low- (LGSOCs) and high-grade serous ovarian carcinomas (HGSOCs).

**Results:**

HGSOC had a significantly higher proportion of advanced stage disease, higher CA125 levels, higher proportion of post-surgery residual disease and higher recurrence or disease progression. WT1 was expressed in 71.4% of LGSOCs and in 57.1% of HGSOCs (*p* = 0.32). Focal and/or complete absence of p53 expression with negative p16 expression was found in 90.5% of LGSOCs, in contrast to the 88.1% of HGSOCs with diffuse or complete absence of p53 expression with positive p16 expression (<0.001). The IHC p53/p16 index and the morphological classification were closely matched (*k* = 0.68). In the univariate analysis, FIGO stage, post-surgery residual disease and histological grade were significantly associated with progression-free survival (PFS) and overall survival (OS). The IHC p53/p16 index was associated only with PFS. WT1 was not associated with PFS or OS. According to the multivariate analysis, advanced FIGO stage and presence of post-surgery residual disease remained independent prognostic factors for worst PFS, however these features had only a trend association with OS.

**Methods:**

21 LGSOC and 85 HGSOC stage I–IV cases were included. The morphological classification was assessed according to the World Health Organization (WHO) criteria. Immunohistochemistry (IHC) was performed in tissue microarray slides. IHC p53/p16 index was compared with the morphological classification.

**Conclusions:**

The IHC p53/p16 index was a good marker for the differentiation of LGSOC and HGSOC, but the morphologic classification showed a better association with survival. FIGO stage and post-surgery residual disease remained the only independent prognostic factors for survival.

## INTRODUCTION

High-grade serous ovarian carcinomas (HGSOCs) comprise the majority of serous ovarian carcinomas (SOCs), as they account for 80%–90% of these tumours and are the most aggressive [1–3]. Advanced cases comprise more than 70% of all HGSOCs, and 5-year overall survival is only 30% for this group of patients [3]. Low-grade serous ovarian carcinomas (LGSOCs), based on their indolent growth pattern, do not respond well to chemotherapy, but they have a better prognosis with significantly longer progression-free survival (PFS) rates [1, 3]. The morphological aspects of SOCs and their histological grades provide important diagnostic information, but immunohistochemistry (IHC) is an important tool that is used in differential diagnosis and in the evaluation of molecular features, which further aids in the characterization of morphology and clinical behaviour [4].

The Wilms Tumor 1 (*WT1*) gene, located on chromosome 11p13, was first identified as the gene responsible for the development of a childhood malignancy [5]. However, *WT1* expression has also been demonstrated in various adult cancers [6–8]. Its location in the female genital tract is usually used to distinguish SOC from other tumour types. Some studies have evaluated the immunoexpression of WT1 and its correlation with prognosis in ovarian cancer [9–13].

*TP53* gene encodes the 53-KDa nuclear protein that is responsible for maintaining the integrity of the genome via the induction of cellular apoptosis in cases of DNA damage [14]. Mutations in the *TP53* gene may be suggested by IHC criteria. *TP53* gene mutations are present in nearly 100% of HGSOCs [3]. Diffuse and strong nuclear expression or complete lack of expression (null type) are associated more with *TP53* mutations, whereas focal expression (wild type) is suggestive of the absence of mutations in HGSOC [4, 15, 16]. LGSOCs are categorized by their low number of genetic mutations; for instance, *TP53* mutations are almost never present in these tumours [3, 17].

p16 is a protein encoded by the *CDKN2A* tumour suppressor gene. The p16 protein performs an important role in cell cycle regulation by decelerating cell progression from G1 to S phase [18]. Approximately 60%–80% of HGSOCs show diffuse p16 staining, [16, 19, 20] for this reason p16 in association with p53, are used as IHC markers in the differential diagnosis of SOCs [4, 21].

Recently, Köbel *et al*. [4] proposed an association among WT1, p53 and p16 immunohistochemical expression in the differential diagnosis between LGSOC and HGSOC.

In the present study, we aimed to examine the diagnostic value of these markers, because interobserver variability occurs in clinical practice, in the distinction between LGSOC and HGSOC [4]. We were then able to verify the immunohistochemical expression of WT1, p53 and p16 in SOCs, in which the histological evaluation was clear, in a specialized gynaecological centre in Brazil. Moreover, we also determined the prognostic value of these markers.

## SUBJECTS AND METHODS

### Participants and tissue specimens

For this retrospective cohort study, we retrieved consecutive formalin-fixed paraffin-embedded (FFPE) tissue samples and the accompanying clinical files of 138 women who were diagnosed and treated at the Women’s Hospital of Campinas State University, Campinas, Brazil, from 1994 to 2013 and who were followed-up until 2016. The local institutional ethics committee (CEP 1086/2009) approved this study. All pathological specimens collected during primary surgery or before neoadjuvant chemotherapy were analysed by an expert gynaecological pathologist (L.A.) according to the guidelines of the World Health Organization (WHO) International Classification of Ovarian Tumours [21]. Stage was classified according to FIGO recommendations and was updated and revised during data collection [22]. Exclusion criteria were as follows: second primary cancer (2 women), no available FFPE tissue sample before chemotherapy (22 women), misdiagnosis (2 women) and missing files (6 women). FFPE tissue samples from the remaining 21 cases of LGSOC and 85 cases of HGSOC with complete data were selected. The data were obtained from each patient’s files. Women underwent chemotherapy regimens that consisted of carboplatin and either paclitaxel or cyclophosphamide according to the service protocols. For both the PFS and OS, the time was estimated in months, from the date of diagnosis to the last follow-up visit, recurrence or any cause of death [23]. The platinum response was classified as recommended by Patch *et al.* [24].

### Tissue microarray (TMA)

Slides from the original paraffin blocks were stained with haematoxylin and eosin (H&E) and were reviewed so that representative areas of the tumour could be identified. Tissue microarray blocks (TMA, Beecher Instruments Microarray Technology, Silver Spring, CA, USA) were constructed using two samples from each case. Sections were obtained from each TMA and were placed on electrically charged slides for all IHC procedures.

### Immunohistochemistry (IHC)

After initial deparaffinization, endogenous peroxidase activity was blocked with 0.3% hydrogen peroxide. The sections were then microwaved in 10 mM citrate buffer (pH 6.0) or Tris-EDTA to unmask the epitopes. The slides were incubated with the following primary antibodies according to optimized protocols: monoclonal mouse anti-human Wilms Tumor 1 (WT1) protein (clone 6F-H2; DAKO Corporation, Carpinteria, CA, 1:100), monoclonal mouse anti-human p53 protein (Clone DO-7, DAKO Corporation, Carpinteria, CA, 1:500), and monoclonal mouse anti-p16 (CINtec^®^ histology V-Kit, clone E6H4, Roche mtm laboratories AG, Germany). The peroxidase-labelled polymer ADVANCE^™^ HRP Detection System (Dako) was applied for 30 minutes at room temperature. DAB chromogen substrate (3-diaminobenzidine, Sigma-Aldrich, St. Louis, MO, USA) was applied, at a proportion of 0.06 g to 100 mL of PBS, 500 μL hydrogen 3% peroxide and 1 mL dimethyl sulfoxide at 37° C for 5 minutes. The slides were subsequently washed in water, counterstained in haematoxylin, dehydrated and mounted. Tissue samples with adequate immunoreactivity were used as positive controls for each antibody. Negative controls were produced by omission of the primary antibodies.

### Evaluation of the immunohistochemical reactions

A single gynaecological pathologist (L.A.) with expertise in ovarian cancer, who was blinded to the clinical and pathological data, scored the samples. Two TMA sets of each tumour component were used for each marker, i.e., each tumour area was assessed twice. The reactions were evaluated according to the percentage of positive cells. In a *post hoc* analysis, if scores differed in the two analyses, the stronger expression was considered.

**Figure 1:**
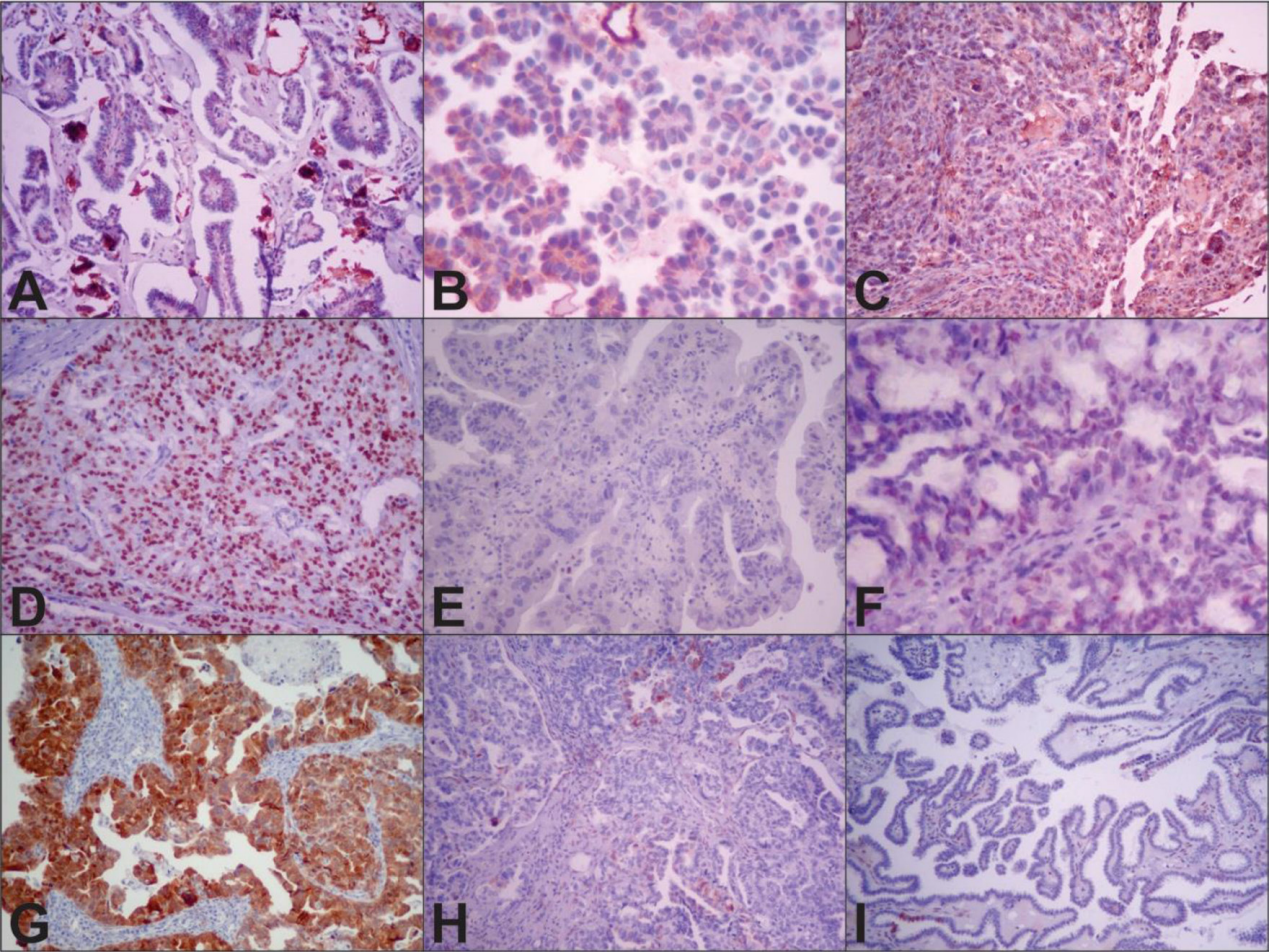
Representative WT1, p53 and p16 immunohistochemical expression in low- (LGSOC) and high-grade serous ovarian carcinoma (HGSOC) Notes: (**A**) positive expression of WT1 in LGSOC (10×); (**B**) negative expression of WT1 in LGSOC (40×); (**C**) positive expression of WT1 in HGSOC (10×); (**D**) diffuse nuclear expression of p53 in HGSOC (10×); (**E**) complete absence of p53 (null type) in HGSOC(10×); (**F**) focal nuclear expression of p53 (wild type) in LGSOC (40×); (**G**) positive nuclear and cytoplasmic expression of p16 in HGSOC (10×); (**H**) negative (focal expression) of p16 in HGSOC (10×); (**I**) negative (expression in only a few cells) of p16 in LGSOC (10×).

Nuclear WT1 protein expression was analyzed in each case, and the percentages of cells with nuclear staining were estimated independently of intensity. Cases with ≥1% positive tumour nuclei were considered positive, and those with zero or less than 1% were considered negative [25]. Nuclear p53 protein expression was analysed in each case, and the percentages of cells with nuclear staining were estimated as follows: complete absence, focal nuclear staining (≥1% and <70% of tumour cells), and diffuse nuclear staining (≥70% of tumour cells) [4, 26, 27]. Cytoplasmic and nuclear p16 staining were described as follows: expression was negative when <10% of cells were stained, if no cells were stained or if cells were stained with low intensity; focal expression when between 10% and 90% of cells were stained; and diffuse when ≥ 90% of the cells were stained [4]. Cases in which > 90% of cells were stained were considered positive, and cases in which <90% of the cells were stained were considered negative (Figure 1).

We also performed a combined IHC classification proposed by Köbel *et al* [4]., the IHC p53/p16 index. According to this classification, a low-grade pattern was defined as p53 staining in ≥1% and <70% of cells and/or the complete absence of p53 associated with p16 staining in <90% of cells. A high-grade pattern was defined as p53 staining in >70% of cells (independent of p16) or the complete absence of p53 staining associated with p16 staining in >90% cells. This approach was used to distinguish SOC on the exclusive basis of IHC, regardless of the morphological classification.

### Statistical analyses

Differences between groups were analysed using the Chi-square test or Fisher’s exact test. Continuous data were analysed using the Mann-Whitney test. Weighted Kappa was calculated to evaluate the IHC p53/p16 index with the morphological classification of the tumors. Progression-free survival (PFS) was measured from the time of diagnosis until relapse, progressive disease, or last follow-up, and overall survival (OS) from the time of diagnosis until any cause of death or last follow-up. PFS and OS probabilities were estimated by the Kaplan-Meier method, and curves were compared by the log-rank test. The Cox hazards model was used to identify variables that predicted PFS and OS. Variables for which *p* ≤ 0.10 in the univariate Cox analysis were included in the multivariate Cox analysis. Differences were significant when *p* < 0.05.

## RESULTS

In this study, the main clinical and pathological features of 106 cases of SOC were recorded. Briefly, the sample was composed of 85 HGSOCs (80.2%) and 21 (19.8%) LGSOCs. Women with HGSOC accounted for a significantly higher proportion of advanced stage disease (80.0% vs 42.9%, *p <* 0.001), presented with higher CA125 levels (median 954 U/ml vs 98 U/ml, *p* < 0.001), had a higher rate of post-surgery residual disease (53.0% vs 19.1%, *p* < 0.01) and had higher recurrence/progression rates (62.3% vs 23.8%, *p* = 0.001) compared with women with LGSOC. No association was observed between LGSOC and HGSOC and age, menopausal status or response to platinum-based chemotherapy (Table 1). In our study, patients with LGSOC were slightly older than their counterparts harboring HGSOG. This finding contrasts to what has been reported in the literature, which tend to concur in that HGSOC has a tendency to affect women older than those harboring LGSOC [3, 24, 28].

**Table 1:** Clinical features of women with low- and high-grade serous ovarian carcinomas

Clinical features	Low-grade serous ovarian carcinoma (LGSOC)	High-grade serous ovarian carcinoma (HGSOC)	
	*n* (%)	*n* (%)	*p* value
	21 (19.8)	85 (80.2)	
**Age median (range), in years**	55 (26–78)	59 (19–85)	0.83
**Menopausal status**			
Pre-menopausal	6 (28.6)	20 (23.5)	
Post-menopausal	15 (71.4)	65 (76.5)	0.78
**FIGO staging**			
I + II	12 (57.1)	17 (20.0)	
III + IV	9 (42.9)	68 (80.0)	**<0.001**
**CA125 median** (1st; 3rd quartile) in U/ml	98 (21; 378)	954 (194; 2248)	**<0.001**
**Post-surgery residual disease**			
No	17 (80.9)	40 (47.0)	
Yes	4 (19.1)	45 (53.0)	**<0.01**
Response to platinum-based chemotherapy^*^			
Platinum-sensitive	10 (91.0)	41 (63.1)	
Platinum-refractory/resistant	1 (9.0)	24 (36.9)	0.09
Recurrence/disease progression			
No	16 (76.2)	32 (37.7)	
Yes	5 (23.8)	53 (62.3)	**0.001**

FIGO: The International Federation of Gynecology and Obstetrics; LGSOC: low-grade serous ovarian carcinoma; HGSOC: high-grade ovarian serous carcinoma; ^*^6 LGSOC patients did not undergo chemotherapy because they were stage I FIGO and 8 HGSOC patients did not undergo chemotherapy because they died; LGSOC and HGSOC women with acquired resistance were not included in this analysis; statistically significant differences are in bold, *p* values were calculated using the Chi square/Fisher exact test or the Mann-Whitney test.

WT1 was expressed in 71.4% of LGSOCs and in 57.1% of HGSOCs, and no significant difference was found in the expression of WT1 in these two tumour types (*p* = 0.32). p53 expression was diffuse in 68.2% of cases, was completely absent in 30.6% (totalling 98.8% of cases) and was focal in 1.2% of HGSOCs, compared with LGSOCs, which demonstrated diffuse expression in 9.5%, complete absence in 81.0% and focal expression in 9.5% (*p* < 0.0001). p16 was expressed in 58.5% of HGSOC samples compared with 9.5% of LGSOC samples (*p* < 0.001). Table 2 shows that the IHC p53/p16 index and the morphological classification are closely matched. It is therefore possible to infer that both classifications can subdivide two distinct SOC subtypes (*p* < 0.0001). Only two samples that were morphologically classified as LGSOC (9.5%) were also classified as HGSOC by the IHC p53/p16 index. On the contrary, 11.9% of HGSOCs that were classified as such on the basis of morphology, were reclassified as LGSOCs by the IHC p53/p16 index. When the IHC p53/p16 index and morphologic classification were compared, the kappa Cohen coefficient was moderate (*k* = 0.68). A higher expression of p16 was seen in FIGO stage III and IV disease compared with FIGO stage I and II disease, and no difference was observed in p53 and WT1 expression according to stage (data not shown).

**Table 2:** Comparison of tumour marker expression in low- and high-grade serous ovarian carcinomas according to morphological classification

Immunohistochemistry expression	Low-grade serous ovarian carcinoma (LGSOC) *n* (%)	High-grade serous ovarian carcinoma (HGSOC) *n* (%)	^1^*p* value
WT1 expression^*^			
Negative (complete absence to <1%)	6 (28.6)	36 (42.9)	
Positive (≥1%)	15 (71.4)	48 (57.1)	0.32
**p53 expression**			
Focal (≥1% and <70%)	2 (9.5)	1 (1.2)	
Complete absence	17 (81.0)	26 (30.6)	
Diffuse (≥70%)	2 (9.5)	58 (68.2)	**<0.0001**
**p16 expression†**			
Negative (complete absence to < 90%)	19 (90.5)	34 (41.5)	
Positive (≥90%)	2 (9.5)	48 (58.5)	**<0.001**
**IHC p53/p16 index**			
Low-grade pattern (p53 staining in ≥1% and <70% and/or p53 complete absence + p16 <90%)	19 (90.5)	10 (11.9)	^2^**k = 0.68**
**High-grade pattern** (p53 ≥70% or p53 complete absence + p16 ≥90%)	2 (9.5)	74 (88.1)	***p*** < 0001

LGSOC: low-grade serous ovarian carcinoma; HGSOC: high-grade ovarian serous carcinoma; ^*^1 woman with HGSOC and ^†^3 women with HGSOC had missing data due to exhaustion of tumour material in the paraffin blocks; statistically significant differences are in bold; ^1^*p* values were calculated using the Chi-square or Fisher exact test; ^2^Kappa Cohen.

For the entire cohort, the median follow-up duration was 56 months (range: 1–213 months). At 60 months of follow-up, the PFS and OS were 37.1% and 50.9%, respectively. The PFS and OS were 73.0% and 83.1%, respectively, in women with LGSOC and 29.1% and 43.8%, respectively, in women with HGSOC (data not shown).

**Figure 2:**
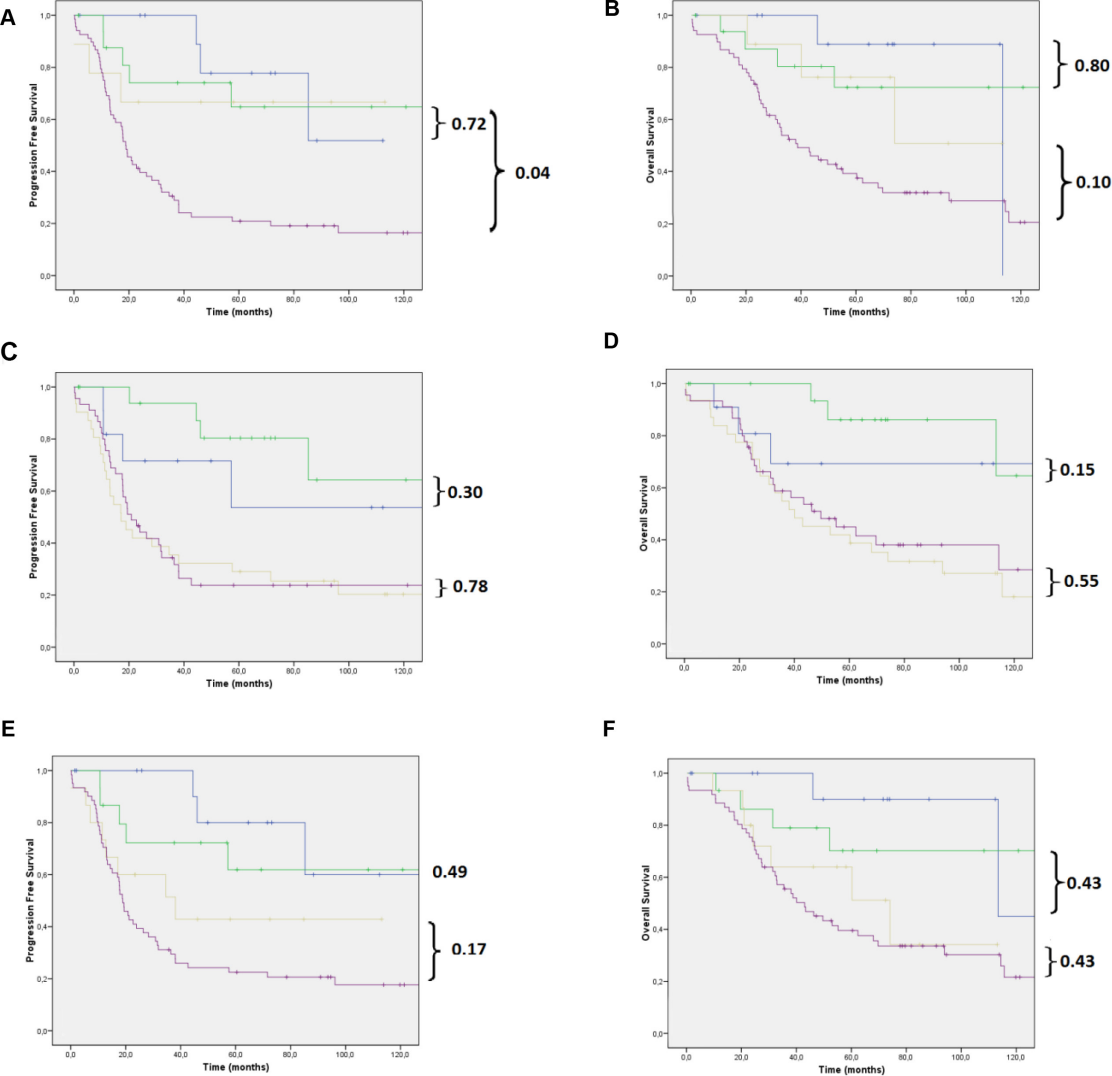
(**A**) PFS and (**B**) OS of women with LGSOC and HGSOC in initial and advanced disease stages based on morphological differentiation (WHO); (**C**) PFS and (**D**) OS of women with LGSOC and HGSOC in cases of negative and positive WT1 expression; (**E**) PFS and (**F**) OS of women with LGSOC and HGSOC in initial and advanced disease stages based on the immunohistochemical p53/p16 algorithm. All our analyses were performed in patients with stage I + II (blue and green lines) or in patients with stage III + IV (yellow and purple lines) disease. Note: Blue line: LGSOC stage I + II; green line: HGSOC stage I + II; yellow line: LGSOC stage III + IV; purple line: HGSOC stage III + IV.

According to the univariate analysis, advanced FIGO stage, the presence of post-surgery residual disease and high tumour grade (morphological classification) were significantly associated with worse PFS and OS. The IHC p53/p16 index was associated with worse PFS (HR = 2.19; 95% CI: 1.10–4.34) but only marginally with OS (HR = 1.99; 95% CI: 0.98–4.08) (Table 3). WT1 expression was not associated with PFS or OS. After the multivariate analysis, advanced FIGO stage and presence of post-surgery residual disease remained independent prognostic factors for PFS. Women with advanced FIGO stage (III + IV) and the presence of post-surgery residual disease had a 2.87 (95% CI: 1.15–7.18) and a 2.04 (95% CI: 1.12–3.71) greater chance, respectively, of progression compared with women with FIGO stage I + II and the absence of post-surgery residual disease. There was a trend association between FIGO staging (HR = 2.28; 95% CI: 0.96–5.39; *p* = 0.06) and post-surgery residual disease (HR = 1.73; 95% CI: 0.96–3.13; *p* = 0.06) with OS in a multivariate model. The IHC p53/p16 index was not an independent prognostic factor for either PFS or OS (Figure 2).

**Table 3:** Survival analysis considering key clinical and pathological features

Clinical features	Progression-free survival			Overall survival		
	Number of recurrence or disease progression/total	HR (95% CI)	*p* value	Number of deaths/total	HR (95% CI)	*p* value
**FIGO staging**						
FIGO I+II	6 / 29	Reference		7/29	Reference	
FIGO III+IV	52 / 77	4.87 (2.08–11.38)	**<0.001**	50/77	3.48 (1.57–7.71)	**0.002**
Post-surgery residual disease						
No	20 / 57	Reference		20/57	Reference	
Yes	38 / 49	3.39 (1.96–5.88)	**<0.001**	37/49	2.58 (1.49–4.45)	**0.001**
**Histological grade**						
Low histological grade	5 / 21	Reference		5/21	Reference	
High histological grade	53 / 85	3.62 (1.44–9.09)	**0.006**	52/85	2.95 (1.17–7.41)	**0.02**
**WT1 expression**						
Negative (complete absence to <1%)	24 / 42	Reference		27/42	Reference	
Positive (≥1%)	33 / 63	1.19 (0.70–2.01)	0.51	29/63	1.45 (0.86–2.46)	0.16
**IHC p53/p16 index**						
Low-grade pattern (p53 staining in ≥1% and <70% and/or p53 complete absence + p16 <90%)	10/29	Reference		9/29	Reference	
High-grade pattern (p53 ≥70% or p53 complete absence + p16 ≥90%)	48/76	2.19 (1.10–4.34)	**0.02**	47/76	1.99 (0.98–4.08)	0.05

FIGO: The International Federation of Gynecology and Obstetrics; PFS: progression-free survival; OS: overall survival; CI: Confidence interval; HR: Hazard ratio; statistically significant differences are indicated in bold. In the multivariate analysis, FIGO staging and histological grade were significantly associated with PFS; FIGO III + IV had an HR = 2.87 (95% CI: 1.15–7.18) and presence of post-surgery residual disease had an HR = 2.04 (1.12–3.71) and were more likely to progress compared with FIGO I + II and absence of post-surgery residual disease. There was a trend association between FIGO staging (HR = 2.28; 95% CI: 0.96–5.39; *p* = 0.06) and post-surgery residual disease (HR = 1.73; 95% CI: 0.96–3.13; *p* = 0.06) with OS in a multivariate model.

## DISCUSSION

In women with clinically advanced ovarian carcinoma, the current treatment consists of surgical cytoreduction combined with adjuvant or neoadjuvant paclitaxel and carboplatin chemotherapy [29]. Optimal cytoreduction surgery, that is, absence of macroscopic residual disease, is the most important prognostic factor related to survival when associated with platinum-based chemotherapy [30, 31].

The two types of SOC harbour different molecular abnormalities and have different clinical courses [3]. These data from the literature were confirmed in our study. Women with HGSOC represented a worse prognosis, and higher CA125 levels compared with women with LGSOC.

We aimed to analyse the expression of WT1, p53 and p16 in LGSOC and HGSOC by IHC and to compare this expression with the pathological/clinical features of the tumours and disease outcomes. We observed that the IHC p53/p16 index has a good association with the histopathological morphological classification. In the univariate analysis, the IHC p53/p16 index was associated only with PFS. After the multivariate analysis, FIGO stage remained the only independent prognostic factor for survival.

In the female genital tract, WT1 expression is usually used to distinguish SOCs from other ovarian tumour types. In a recent review, Köbel *et al.* [4] affirm that WT1 expression suggests SOC, considering that approximately 10% of HGSOCs can be negative. In our study, approximately 60% of all SOCs expressed WT1 according to the same method described above [4]. In the current study, WT1 expression was lower than what has been reported in most papers in the recent literature, which might be explained by the irregular staining of tissue for WT1, additionally, the TMAs used may not contain the most immunoreactivity areas of the tumours [9, 32, 33]. TMA analysis pose a few challenges in terms of choosing tumor areas representative of the most significant biological processes taking place during tumor formation and progression. To ensure good hot spot selection, having an experienced pathologist in mandatory. Fortunately, we have a team of seasoned pathologists, who took part in several studies using TMA and developed a vast expertise in selecting tumor areas for TMA assembly. These issues are now dealt with in the discussion. We cannot ascribe the unexpected findings pertaining to WT1 expression to a faulty TMA hotspot selection bias.

In our study, p53 expression was diffuse in 68.2% and was completely absent (null type) in 30.6% of women with HGSOCs (totalling 98.8% of cases). Our results are similar to those of a previous study, which considered p53 expression by IHC (*TP53* mutations are present in nearly 100% of HGSOCs) [3, 26, 34]. p16-positive IHC expression (i.e., ≥90% expression) was observed in 58.5% of women with HGSOC, which is consistent with what has been reported in the literature [4, 21].

Recently, Köbel *et al.* [4] concluded that their IHC p53/p16 index matched the standard pathological categorization of SOC and was reproducible. The IHC p53/p16 index aims to differentiate between LGSOC and HGSOC using p53 and p16 IHC in WT1-positive samples. LGSOCs are characterized by the focal expression of both p53 and p16, whereas HGSOCs are defined as tumours with diffuse p53 expression or complete absence of p53 expression (null type) associated with diffuse p16 expression. In our study, only 1 case presented focal expression of both markers. This prompted us to also consider samples with focal and/or complete absence of p53 staining associated with negative p16 expression as LGSOC [4]. In our study, we observed a close match between the morphological classification recommended by the WHO and the IHC p53/p16 index, principally in women with HGSOC.

*TP53* alterations are associated with high rates of tumour cell proliferation, but the association between p53 expression and patient prognosis remains controversial [35]. In malignant tumours, p16 overexpression appears to be a mechanism by which the uncontrolled proliferation caused by failure of the Rb pathway can be arrested [36]. In our study, the IHC p53/p16 index was not significantly associated with survival.

Some important limitations of our research should be highlighted. First, our IHC evaluation was performed in TMA samples, and it is known that large ovarian cancers can show regional variability in the expression of protein markers. Indeed, IHC analyses were performed after a long period of time which means that the quality of the paraffin-embedded tissue might have been compromised. Second, the same cases that were included in our study were diagnosed almost 20 years ago. Herein, we divided our casuistry based on the diagnostic data: 1994 to 2003 (*n* = 61) and 2004 to 2013 (*n* = 45). No difference was observed in terms of the clinical features or survival (data not shown). Finally, our ratio of LGSOC to HGSOC is higher than that reported in the literature. This might be expected to occur once the majority of HGSOC cases present with advanced FIGO stages and are treated with neoadjuvant chemotherapy; these cases would thus be excluded from the study sample.

## CONCLUSIONS

The IHC p53/p16 index and the morphological classification are closely matched, and the IHC p53/p16 index seems to be a good marker for the differentiation of LGSOC and HGSOC. However, the WHO morphologic classification showed a better association with survival. FIGO stage and post-surgery residual disease remained an independent prognostic factors for PFS but only a trend association with OS. However, the IHC p53/p16 index was not an independent prognostic factor for either PFS or OS.

In the evaluation of a new case of SOC, the initial assessment should be performed according to WHO morphologic criteria. In doubtful cases, IHC should be performed to determine the IHC p53/p16 index. Thus, p53 and p16 expression have an important role in the routine differential diagnosis of ovarian carcinoma.
